# Crustal CO_2_ contribution to subduction zone degassing recorded through calc-silicate xenoliths in arc lavas

**DOI:** 10.1038/s41598-019-44929-2

**Published:** 2019-06-19

**Authors:** Sean Whitley, Ralf Gertisser, Ralf Halama, Katie Preece, Valentin R. Troll, Frances M. Deegan

**Affiliations:** 1 School of Geography, Geology and the Environment, Keele University, Keele, ST5 5BG UK; 2 Department of Geography, College of Science, Swansea University, Swansea, SA2 8PP UK; 3 Section for Mineralogy, Petrology and Tectonics (MPT), Department of Earth Sciences, Uppsala University, 752 36 Uppsala, Sweden; 4 Faculty of Geological Engineering, Universitas Padjajaran (UNPAD), Bandung, Indonesia

**Keywords:** Geochemistry, Volcanology

## Abstract

Interaction between magma and crustal carbonate at active arc volcanoes has recently been proposed as a source of atmospheric CO_2_, in addition to CO_2_ released from the mantle and subducted oceanic crust. However, quantitative constraints on efficiency and timing of these processes are poorly established. Here, we present the first *in situ* carbon and oxygen isotope data of texturally distinct calcite in calc-silicate xenoliths from arc volcanics in a case study from Merapi volcano (Indonesia). Textures and C-O isotopic data provide unique evidence for decarbonation, magma-fluid interaction, and the generation of carbonate melts. We report extremely light δ^13^C_PDB_ values down to −29.3‰ which are among the lowest reported in magmatic systems so far. Combined with the general paucity of relict calcite, these extremely low values demonstrate highly efficient remobilisation of crustal CO_2_ over geologically short timescales of thousands of years or less. This rapid release of large volumes of crustal CO_2_ may impact global carbon cycling.

## Introduction

Crustal magma-carbonate interaction has been suggested as a process that may dominate the CO_2_ output in several volcanic arcs^1,2^ and a possible source of magmatic carbonate melts^3–5^. Direct evidence for this process often remains elusive, but the occurrence of calc-silicate (skarn) xenoliths in the eruptive products of some active volcanoes^6–9^ provide a unique opportunity to study high temperature magma-carbonate interaction, and the subsequent effects on the host magmatic system. Recent work on such xenoliths has additionally linked magma-carbonate interaction to influencing eruptive dynamics via volatile exsolution^7,10–12^, and driving magmatic differentiation trends towards highly desilicated potassic compositions^13,14^.

Carbon and oxygen isotopes are powerful tracers of fluid-rock interaction processes during contact metamorphism of carbonates, where skarn rocks form via reactions between magmatic fluids and carbonate country rocks^15–18^. Sedimentary carbonates are isotopically distinct from mantle-derived igneous rocks^15^, allowing for quantification of chemical exchange during magma-carbonate interaction. Decarbonation reactions release CO_2_ that is enriched in ^13^C and ^18^O into the magmatic system^19^, depleting the carbonate protolith in these isotopes, following batch or Rayleigh fractionation trends^15^. However, some skarns show additional isotopic modifications that cannot result from equilibrium decarbonation reactions alone, but instead require mixing with magmatic fluids to produce values in near-exchange equilibrium with the adjacent magmatic system^16,20–22^. The degree of decarbonation and CO_2_ released into the magmatic system and ultimately the atmosphere, and the extent of fluid modification of the isotopic signature, can be quantified utilising coupled carbon and oxygen isotopes. In addition, interaction with meteoric water and secondary alteration can usually be distinguished using this approach^16^.

Merapi is Indonesia’s most active volcano, characterised by periods of lava dome growth punctuated by dome failure producing pyroclastic density currents, and intermittent explosive events^23^. Compositionally the eruptive products are restricted to high-K basalt to basaltic andesite^23^. The upper crust underlying Merapi consists of a 8–11 km thick unit of Cretaceous to Cenozoic limestones, marls and volcaniclastic deposits^24^, and is found as thermally metamorphosed xenoliths within the eruptive deposits^7,12,25,26^. These xenoliths testify to prevalent magma-carbonate interaction^7,26^, a process that is ongoing and occurs at rapid syn-magmatic timescales^11,12^. Previous work at Merapi has focused on radiogenic (^87^Sr/^86^Sr) and stable (δ^13^C, δ^18^O) isotope analysis of bulk xenoliths and mineral separates of the most abundant calc-silicate mineral phases (wollastonite, diopside)^7,12,25,26^, highlighting the significance of crustal contamination in the genesis of Merapi magmas, and a role of magma-carbonate interaction for enhancing eruption explosivity^12,26–28^. Some of these Merapi xenoliths retain remnant calcite with distinct textural types, which provide an exceptional opportunity to gain novel insights into magma-crustal interaction processes. Our micro-analytical approach allows, for the first time, a detailed assessment of the roles of decarbonation, interaction between carbonates and magmatic fluids, carbonate melt generation, and crustal volatile release. We demonstrate that highly efficient decarbonation of carbonate wallrock at Merapi produces extremely negative calcite δ^13^C values during skarn formation in some xenoliths, whereas others bear contrasting evidence of interaction between carbonate and magmatic fluids. This combination of processes documents fast and highly efficient, rapid liberation of crustal CO_2_ into the atmosphere.

## Results

### Petrography of calc-silicate xenoliths and calcite types

Calc-silicate xenoliths are ubiquitous in the studied 1994 to 2010 Merapi dome lavas and can be divided into two groups on the basis of distinct textures and mineralogical assemblages: magmatic skarns and exoskarns (c.f.^6^; Supplementary Table 1). Magmatic skarn xenoliths contain abundant glass that is CaO-enriched (1 to 12 wt%) relative to host lava dacite groundmass glasses, and dominantly comprise wollastonite which contains numerous CaO-enriched melt inclusions compositionally similar to the contaminated groundmass glass. These wollastonite-hosted melt inclusions and additional Fe-rich growth rims on wollastonite crystals testify to crystallisation from a melt that has assimilated a significant quantity of CaO (c.f.^29^). These xenoliths typically show a general rim-core zoning sequence (idealised): lava - clinopyroxene - plagioclase + clinopyroxene - clinopyroxene - glass - wollastonite core, with additional glass found in varying quantities in each zone. Vapour-rich CO_2_ fluid inclusions are common within wollastonite crystals. Exoskarn xenoliths are holocrystalline, granular, and primarily composed of Ca-Tschermak’s component (CaAlAlSiO_6_) enriched clinopyroxene (fassaite), wollastonite, plagioclase and grossular-andradite garnet, resembling typical skarn assemblages associated with metasomatic alteration^30^. They lack the magmatic skarn xenolith zoning sequence, having only a rim of clinopyroxene at the lava contact.

**Figure 1:**
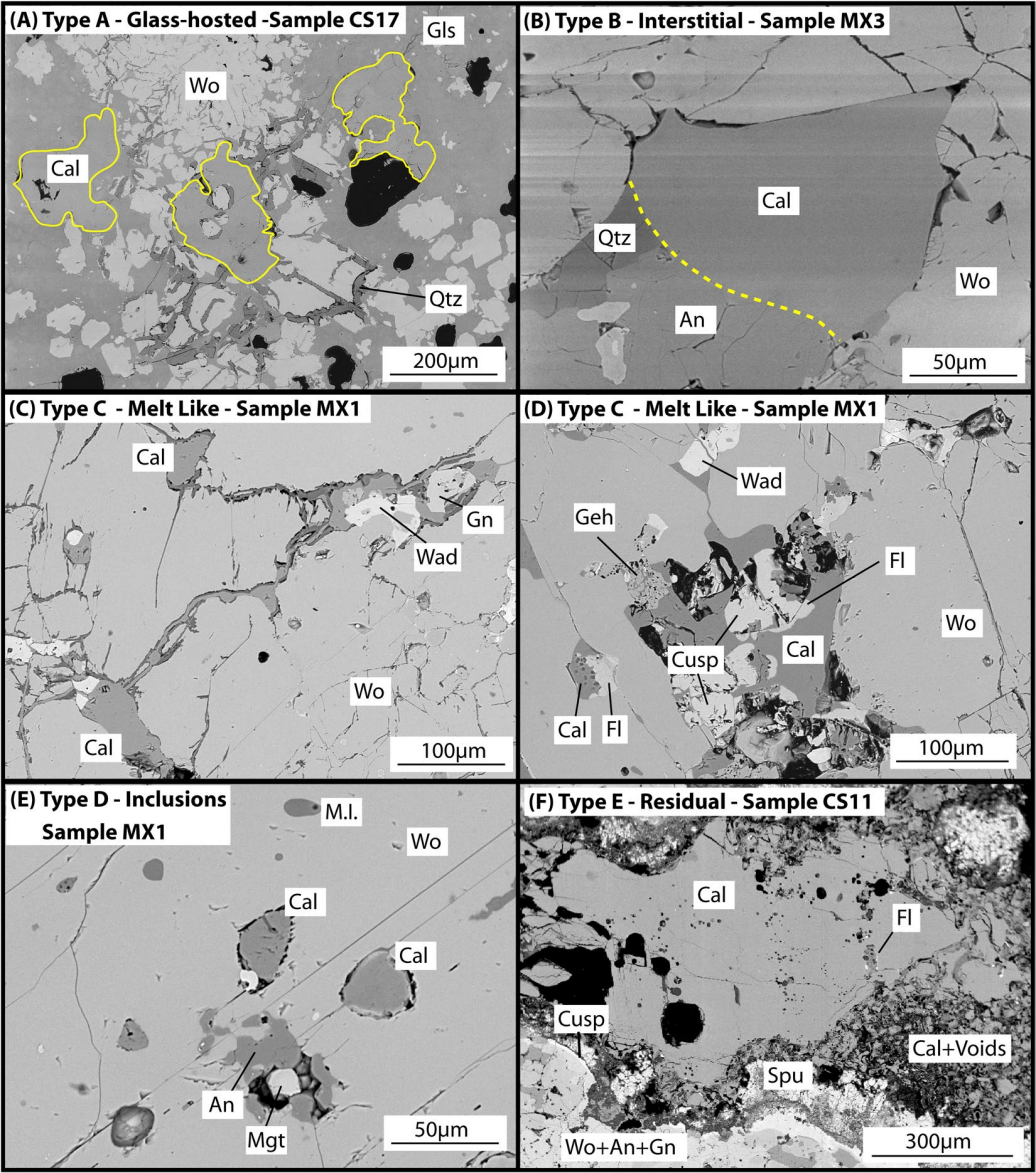
Calcite textural types. Types A-D are from magmatic skarn xenoliths, type E is from exoskarn xenoliths. (**A**) Type A calcites found within the glass band of magmatic skarn xenoliths. Yellow lines highlight the glass-calcite boundary due to poor backscatter contrast. (**B**) Type B interstitial calcite; small crystals within a decussate wollastonite-dominant xenolith core. (**C**) Type C calcite showing pools of calcite joined by veinlets, with garnet and a phase compositionally similar to wadalite (Ca_12_Al_10_Si_4_O_32_Cl_6_) closely associated. (**D**) Type C calcites showing the close association with F-bearing phases fluorite, cuspidine (Ca_4_Si_2_O_7_F_2_) and the wadalite-like phase. (**E**) Type D calcite inclusions within wollastonite. Note the presence of melt inclusions (M.I.) in wollastonite. (**F**) Type E residual calcite showing the zoned reaction rim, with disaggregated calcite and voids, spurrite, then cuspidine and the wollastonite + anorthite + grossular garnet xenolith assemblage. Accessory fluorite is present at some calcite contacts, and very rare micron-sized xenotime and monazite crystals are present within the calcite. Abbreviations: An - anorthite, Cal - calcite, Cusp - cuspidine, Fl - fluorite, Geh - gehlenite, Gls - glass, Mgt - magnetite, M.I. - melt inclusion, Qtz - quartz, Spu - spurrite, Wad - wadalite-like phase, Wo - wollastonite.

We distinguish five textural types of calcite across both of these xenolith groups (Fig. 1). Each textural type represents a specific process, or combination of processes, operating during magma-carbonate interaction, which C-O isotopes provide a means to quantify. Four calcite types are found within the magmatic skarn xenoliths (types A, B, C, D), and two within the exoskarn xenoliths (types D, E). Type D calcites were only analysed in wollastonite in the magmatic skarn xenoliths due to crystal size constraints. Type A calcites consist of rounded globular calcite grains within the glass-rich xenolith textural zone (Fig. 1A). Type B calcites occur as subhedral crystals (50–100 µm in size) that are found interstitial to wollastonite, and as fractured crystals at vesicle borders within wollastonite-dominant cores (Fig. 1B). Type C calcites are anhedral interstitial crystals (50–100 µm in size) exhibiting a melt-like, infiltrative texture between wollastonite crystals. They are found as thin interconnected veins with a rim of quartz at the wollastonite contact (Fig. 1C,D). These veins form rare ~50 µm pools of calcite, with occasional fluorite crystals nucleating at the edges and around calcite-hosted vesicles. Type D calcites occur as rounded inclusions (<50 µm) in wollastonite and garnet (Fig. 1E). Type E calcites occur exclusively within exoskarn xenoliths as millimetre-sized crystals with vesiculated reaction rims containing spurrite and sulphur and fluorine-enriched phases (Fig. 1F). These calcites contain occasional vesicles and trace amounts of phosphates. The rims are anhedral and intermingle with the void-rich reaction rim.

### Geochemistry of calcite types

**Figure 2:**
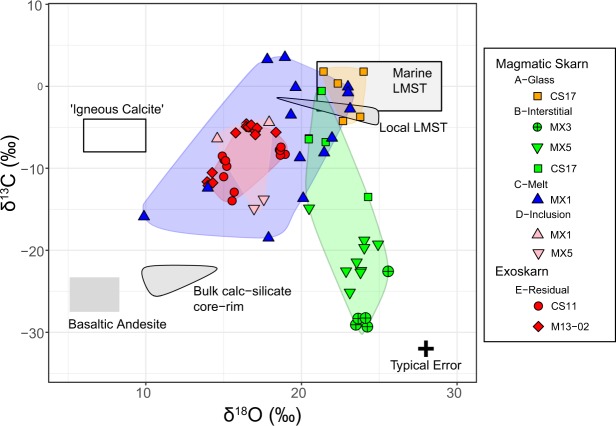
Calcite C-O isotopic compositions. Extra data: Primary ‘igneous’ calcite (calcite in equilibrium with the mantle) field^64^; Cretaceous to Cenozoic marine limestone (Merapi limestone)^24,31^; Bulk calc-silicate and basaltic andesite^12,26^; Local limestone field^12,25,26^. Typical error is 2σ.

The calcites analysed in this study are almost pure CaCO_3_, with MnO, FeO and MgO < 0.17 wt% across all xenoliths (Supplementary Table 2). By contrast, calcite isotopic compositions cover a large range in δ^13^C-δ^18^O values (Table 1, Fig. 2), with different textural groups being compositionally distinct. Assuming a typical marine carbonate protolith (δ^13^C −3 to +3‰, δ^18^O > 25‰^31^) or local Merapi limestone (δ^13^C −5 to −1‰, δ^18^O + 18 to +25‰^12,25,26^) as the starting composition, two compositional trends are defined: (1) a trend towards low δ^13^C-δ^18^O compositions, approaching those of bulk xenolith core/rim mineral separates^12,26^, and (2) a trend towards low δ^13^C with little δ^18^O variation (Fig. 2). The highest δ^13^C-δ^18^O calcites are type A glass-hosted calcites, forming a compositionally tight cluster with δ^13^C between −4.2 and +1.8‰, and δ^18^O ranging from +21.4 to +24‰. Type B interstitial calcites have the largest δ^13^C variation, but a restricted δ^18^O range (δ^13^C −29.3 to −0.6‰, δ^18^O + 20.5 to +25.6‰). Type C melt-like calcites have the widest δ^18^O variation, between +9.9 and +23.1‰, and a large δ^13^C variation, ranging from −18.5 to +3.5‰. Data for Type D calcite inclusions within wollastonite are few, but show a similar compositional range of δ^13^C (−14.9 to −4.4‰), and δ^18^O (+14.6 to +17.9‰) to the type E calcites. Type E-residual calcites in the exoskarn xenoliths, define a tight compositional cluster between δ^13^C of −14 to −4.6‰ and δ^18^O of +13.9 to +19‰.

**Table 1:** Summary of C-O isotopic data for the calcite textural types.

Calcite Type	Xenolith Type	δ^18^O (‰)	δ^13^C (‰)
A – Glass-hosted	Magmatic Skarn	+21.4 to +24.0	−4.2 to +1.8
B – Interstitial	Magmatic Skarn	+20.5 to +25.6	−29.3 to −0.6
C – Melt-like	Magmatic Skarn	+9.9 to +23.1	−18.5 to +3.5
D – Inclusions	Magmatic Skarn	+14.6 to +17.9	−14.9 to −4.4
E – Residual	Exoskarn	+13.9 to +19.0	−14 to −4.6

The full dataset can be found in Supplementary Table 3. 2 σ errors are typically 0.4‰ for oxygen and 0.8‰ for carbon.

## Discussion

We focus this discussion on the processes that produce the compositionally and texturally diverse calcites, and the implications for the Merapi magmatic system and subsequent crustal CO_2_ liberation to the atmosphere (see also Supplementary Discussion for full details). To explain the C-O isotopic variations in calcite from the investigated xenoliths, we consider two distinct end-member processes: (1) decarbonation (devolatilisation) of the originally sedimentary carbonate and/or (2) magmatic fluid-carbonate interaction. We have performed Rayleigh fractionation and fluid mixing modelling, the full details of which are found in the methods.

**Figure 3:**
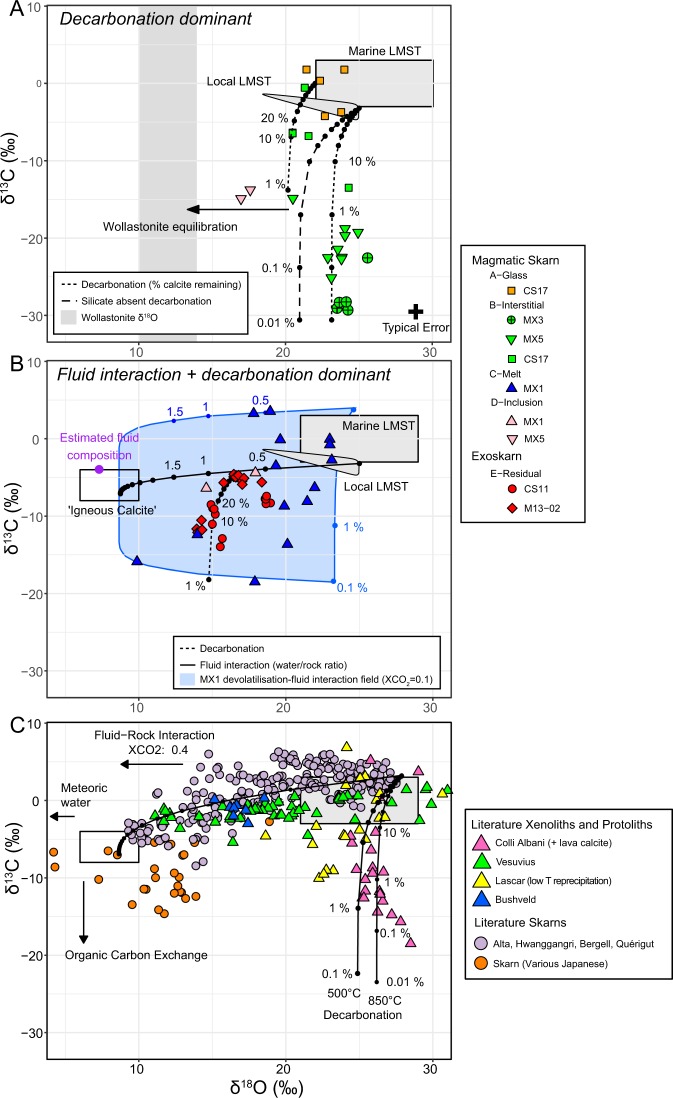
Calcite C-O isotopic variations. (**A**) Calcite types dominated by decarbonation. Points on the decarbonation curves represent percentage of original calcite remaining. (**B**) Calcite types dominated by fluid interaction and decarbonation. Points on the fluid interaction curve represent the fluid-rock ratio. (**C**) Literature calcite and skarn data. Modelling curves are purely for illustration, and no attempt is made in our work to accurately model the literature data. See the respective papers for detail. Fields for marine limestone, local limestone and igneous calcite as in Fig. 2. Extra data: Wollastonite oxygen isotope data^26,35^; Skarn xenoliths^5,8,17,18,22,39,40^; Skarns^20,21,38,42,65,66^.

Pure decarbonation follows a Rayleigh fractionation trend, where ^13^C is preferentially removed in the released CO_2_, lowering the δ^13^C of the residual calcite. There is little change in δ^18^O as the newly formed skarn minerals retain the majority of the oxygen. Type A - glass-hosted calcites have the highest δ^13^C and δ^18^O values (Fig. 2), overlapping marine carbonate compositions, and therefore are the most likely calcites to represent unmodified carbonate compositions at Merapi. The limestones sampled local to Merapi^12,25^ have a slightly lighter δ^13^C composition, therefore we modelled two Rayleigh fractionation curves with both these starting compositions (Fig. 3A). Type B interstitial calcites from two of the magmatic skarn xenoliths (samples MX3 and MX5) follow the decarbonation model closely, showing very high levels of decarbonation (Fig. 3A). The fraction of calcite remaining falls between 0.01 and 0.0001 (Fig. 3A), consistent with the very low (<1 vol%) modal calcite content in these xenoliths. All interstitial Type B calcites are in close association with a wollastonite-dominant mineral assemblage (Fig. 1), indicating that the formation of wollastonite by the reaction $\mathop{CaC{O}_{3}}\limits^{calcite}+\mathop{Si{O}_{2}}\limits^{silica}=\mathop{CaSi{O}_{3}}\limits^{wollastonite}+C{O}_{2}$ is likely to be the key process causing the observed depletions in δ^13^C. Decarbonation can also occur via the silicate-absent reaction $\mathop{CaC{O}_{3}}\limits^{calcite}=\mathop{CaO}\limits^{lime}+C{O}_{2}$. No lime is observed, but the CaO may have transferred to the melt (c.f.^11^), producing the CaO-enriched xenolith glass from which wollastonite precipitated, trapping the observed abundant melt inclusions. The modelled curve for silicate-absent decarbonation (Fig. 3A) is approximately two δ^18^O units lower than most of the values for the interstitial calcite in the magmatic skarns. However, if a higher δ^18^O for the protolith is chosen, this curve could equally well reproduce the calcite C-O isotope compositions in samples MX5 and MX3. A protolith with a higher δ^18^O is not observed in the local literature limestone data, but it is plausible based on marine carbonate rocks having a wide range of δ^18^O values^31^, exceeding +30‰ in some limestones^17,22^. Therefore, we cannot conclusively distinguish between the two processes given the potential isotopic variability in the marine carbonate protolith. The degree of decarbonation is however shown to be extremely high independent of the exact process, as demonstrated by the very low δ^13^C values down to −29.3‰, more than 20 δ^13^C units below the presumed protolith. Hence, the fraction of calcite remaining in these xenoliths is less than 1% and possibly as small as 0.01%. This, in turn, shows that decarbonation is very efficient in the Merapi magmas.

The remaining calcites (Types C, D, E) show significant deviation from modelled decarbonation curves (Fig. 3A). Various studies have shown that the C-O isotopic compositions of carbonates in skarns typically cannot be explained fully by decarbonation, and often requires interaction with magmatic fluids, causing shifts towards magmatic C-O isotopic values^15^. A simple magma-carbonate mixing curve however cannot reproduce the bulk of the data, in contrast to many worldwide skarns^15^ (Fig. 3B,C). The Type C melt-like calcites (Fig. 3B) have a significant spread of data, and show a complex mixing-decarbonation history. A magmatic fluid is required to produce the strong δ^18^O depletions, which is evidenced directly in this sample by the presence of localised areas containing calcite, the F-bearing phases fluorite and cuspidine, and a Cl-bearing phase compositionally similar to wadalite. The presence of a high temperature magmatic brine at Merapi has been previously identified^32,33^, which has interacted with some of the xenoliths. Depletion in δ^13^C requires decarbonation, which may occur during carbonate melting, releasing CO_2_ whilst producing a Ca-rich melt^34^, and/or during the formation of wollastonite via decarbonation reactions. The entire spectrum of type C calcite compositions can then be modelled by a single decarbonation curve and subsequent mixing with a magmatic fluid, or vice versa (Fig. 3B).

Type D wollastonite-hosted calcite inclusions in samples MX5 and MX1 (Fig. 3A,B) can be modelled by a combination of decarbonation followed by interaction with either a fluid, or diffusive equilibration between the calcite inclusion and the host wollastonite crystal. Host wollastonites have δ^18^O values of +10 to +14‰^26,35^, and any equilibration between wollastonite and calcite would cause a large reduction in calcite δ^18^O values (Δ^18^O_calcite-wollastonite_ at 850 °C = 2.2^36^).

Residual type E calcites (Fig. 3B) within the exoskarn xenoliths can be modelled by fluid mixing followed by decarbonation from a local limestone initial composition. Due to the absence of glass, the relatively lower temperatures compared to the magmatic skarn xenoliths constrained from the phase assemblage^37^, and the presence of magmatic volatile-bearing phases (apatite-ellestadite, cuspidine, fluorite, anhydrite), these xenoliths likely represent samples from a greater distance into a zoned carbonate alteration zone around the magma^6,17^. This scenario is similar to zoned contact metamorphic aureoles and exoskarns, where interaction with fluid released from a cooling magma may have a stronger influence than the dominant thermal metamorphic influence adjacent to the magma. The second stage decarbonation trend may have been produced as the sample is entrained in the melt during ascent, as decarbonation is shown to be dominant in the syn-magmatic skarn xenolith samples MX3 and MX5.

In summary, the isotopic variations between calcite types can be attributed to whether a magmatic fluid phase was present during xenolith formation. Type A calcites represent nearly unmodified calcite compositions. C-O isotope systematics of interstitial calcites (type B) in magmatic skarn samples can be explained by decarbonation reactions alone (Fig. 3A). The calcites were affected dominantly by the heat of the magmatic melt. The liberated CaO from decarbonation reacted with the melt SiO_2_ to produce wollastonite, containing abundant melt inclusions and CO_2_-rich fluid inclusions. Type C melt-like calcites show evidence for the presence of a F + Cl-bearing magmatic fluid phase. This magmatic fluid facilitated calcite melting and oxygen isotope exchange. Simultaneous decarbonation reactions occurred forming wollastonite, similar to Type B calcites (Fig. 3A). Wollastonite-hosted calcite inclusions (type D) represent equilibration with their host phase, lowering their δ^18^O values. Type E calcites in the exoskarn xenoliths (samples CS11 and M13–02) lack a silicate glass phase, and have a distinct mineral assemblage, indicating they are located from within an aureole alteration zone around the magma body. Pure thermal decarbonation is likely a less prominent isotopic modification process, and instead a stronger influence of magmatic fluid interaction is recorded (as in skarns^15^). These exoskarns therefore record a pronounced lowering in their calcite δ^18^O values (+13.9 to +19‰) and only moderate changes in δ^13^C (Fig. 3B).

This variety of processes at Merapi contrasts with other reported skarn and skarn xenoliths. Figure 3C shows a selection of oxygen and carbon isotope data from skarn deposits and skarn xenolith suites. Skarns that form in carbonate country rocks adjacent to granitoid intrusions typically show dominantly fluid mixing trends^15,16^, with variation in δ^13^C attributed to a smaller degree of decarbonation. Significant depletions in δ^13^C to below mantle values are often attributed to interaction with organic material such as graphite-bearing wall rocks or a CH_4_-rich fluid^16,38^. Skarn xenoliths from active volcanoes and volcanic areas often exhibit two distinct trends in C-O isotope space. Calcite from Vesuvius^18,22,39^ and Bushveld^40^ skarn xenoliths follow the typical skarn trend, with compositions defining a trajectory towards mantle and carbonatite C-O isotope compositions. Xenoliths and groundmass calcite within lavas from Colli Albani^5,8^, however, show a pure decarbonation trend, with negligible magmatic influence. At Merapi, our type B interstitial calcites show a dominant decarbonation trend, similar to Colli Albani (Fig. 3C), however the extremely low δ^13^C values observed at Merapi (down to δ^13^C = −29.3‰) suggest more extreme degrees of decarbonation. To the best of our knowledge, these calcites record the lowest carbonate δ^13^C values reported from skarns, approaching that of organic carbon. Our data, and the literature whole-rock data^12^, show that low δ^13^C values can be achieved by high temperature decarbonation reactions alone. In the absence of other geological evidence of organic material interaction, this could indicate that other depleted skarns in the literature may reflect similar processes, without the need to involve organic carbon sources. The exoskarn xenoliths from Merapi lack the definitive skarn-like trend shown by Vesuvius and the various skarns associated with granitoid intrusions. Instead, they demonstrate a combination of both trends, lowering δ^13^C values to below typical igneous values, which we interpret as the combined action of decarbonation and interaction with magmatic fluids. Contrasting processes of pure decarbonation and fluid infiltrative isotopic modification have been only rarely described from individual contact metamorphic aureoles, such as the Bufa Del Diente alkali syenite intrusion^41^ and the Quérigut magmatic complex^42^. Xenolith evidence for contrasting processes is also rare, and where present is much less pronounced^18,22^ than that observed at Merapi. This clear record of the strong effects of contrasting processes may be due to the rapid timescales of carbonate assimilation^11,43^ which would hinder preservation of high temperature thermal decarbonation in typical contact aureoles, but are preserved in the xenoliths that record transient snapshots of interaction processes. Additionally, these xenoliths from active volcanic systems record the prograde stage of skarn formation, which is often overprinted or completely replaced by retrograde and alteration facies in *in situ* contact aureoles. Granitic intrusions typically intrude at lower temperatures than basaltic-andesite magmas, therefore the country rock is likely to experience less intense thermal effects, and therefore less decarbonation, compared to Merapi country rock xenoliths that experience temperatures upwards of 925 °C^44^. Furthermore, a cooling granitic body would release large quantities of fluid as magmatic crystallisation progresses, producing a fluid-dominant isotopic signal in the aureole. Indeed, in the Merapi xenoliths, samples that exhibit evidence for a brine phase during formation (i.e. F, Cl and S-rich mineral phases), are the samples that show a shift towards magmatic isotopic values, confirming the presence of a fluid that influenced the xenolith’s isotopic composition.

In addition to modifying the isotopic composition of the calcites, the fluids facilitated carbonate melting, and therefore our xenolith samples provide new evidence that carbonate melts can be produced by interaction between magmas and crustal carbonate^3–5^. Our textural evidence shows calcite with typical melt-like structures that we interpret as having formed from a carbonate melt. A crustal source is evidenced by the calcite isotopic compositions overlapping those of marine carbonates. Some of the analysed melt-like calcites approach the C-O isotope values typical of magmatic mantle derived carbonates, which demonstrates interaction of the crustal carbonate with magmatic fluids (Fig. 3B). The carbonate melts formed in the xenoliths are volumetrically small, as formation of skarn minerals via decarbonation reactions is the dominant process of magma-carbonate interaction at Merapi, and the carbonate melts require a F-rich fluid^45^ and a Ca-rich contaminated magmatic melt to stabilise the carbonate melt within the xenoliths. This restricts the melt locally to the xenoliths as to our knowledge, no carbonate is found within the basaltic-andesite lavas. This is in contrast to examples such as Colli Albani^5,8^ and the Hortavær igneous complex in central Norway^46^, where carbonate derived from country rock assimilation is frequently found in the igneous materials. The xenolith Ca-rich melts are isolated from the host magma by a rim of clinopyroxene and plagioclase, however the low viscosity of carbonate and Ca-contaminated melts^11,47^ may allow release from the xenoliths into the Merapi magma via filter pressing or through xenolith disaggregation. These melts alone are likely to have a limited effect on the bulk magmatic composition due to the small volumes of carbonate melt produced, relative to usage of magmatic elements to form the skarn mineral assemblages. Regardless of the exact mechanism of magma-carbonate interaction (mixing with carbonate melts, skarnification, bulk dissolution), a strong magma-crustal carbonate interaction signature at Merapi is demonstrated by isotopic^7,12,25–27,35^ and trace element^1^ studies, indicating up to 30% contamination with crustal carbonate components.

Our modelling results, and the general paucity of relict calcite in the xenoliths, demonstrate that magma-carbonate interaction at Merapi is very efficient at remobilising crustal CO_2_ into the magmatic system and ultimately the atmosphere. Crustal carbonate assimilation has been shown to be an important contributor to CO_2_ output at Merapi^11,12,48^ and a widespread occurrence in arc volcanoes, which may even dwarf contributions from source contamination^1,2^. We have used a mass balance model^49^ (see methods) to place constraints on the amount of crustal CO_2_ produced at Merapi. We find that 24–56% of CO_2_ emissions are crustal derived during quiescence, and 41–95% during eruptive periods. Contributions to crustal CO_2_ release come from direct carbonate assimilation into magma, thermal carbonate breakdown, and metasomatic alteration of the wall rock. Modelling the CO_2_ released from a contact aureole^22^ produced by a magma reservoir similar in size to that which produced the 2010 eruption of Merapi^50^ (see methods) indicates that 9.5 × 10^9^ to 1.8 × 10^11^ kg of crustal CO_2_ could be released from the limestone surrounding the reservoir. Merapi degases approximately 4–4.6 × 10^5^ kg of CO_2_ per day^51^, of which we calculate 24–56% may be crustal derived during quiescence (see above). This means that the total CO_2_ that could be released from the aureole of a reservoir similar in size to that of the 2010 eruption could occur rapidly over 119 to 4960 years. This timescale may be shortened by punctuated periods of eruptive activity that increase carbonate interaction^12,28^. To simulate a maximum CO_2_ output over the lifespan of Merapi, we can consider multiple reservoirs intruded into the carbonate at variable depths, which will cause much larger volumes of CO_2_ to be released. Fully decarbonating a vertical limestone cylinder from the surface to the base of the limestone at ~10 km^24^ with a 1 km radius, corresponding to the estimated 2010 eruption reservoir width +50%, shows that up to 3.8 × 10^13^kg of CO_2_ could be released. Although Merapi is currently considered a relatively low global CO_2_ emitter^52^, our calculations, and documented strongly degassed syn-magmatic xenoliths, show that crustal CO_2_ liberation can be temporarily variable with potentially large amounts released during eruptive episodes compared to periods of overall quiescence^11,12^. We assume that carbonate-interacting arc systems follow similar patterns worldwide, and probably over geological time too.

Periods of global warming in the Earth’s past, such as the Cretaceous hothouse and the Paleocene-Eocene Thermal Maximum (PETM) have been discussed in the context of excess atmospheric carbon originating from either organic carbon release^53^, intense volcanism^54^ and/or increased magma-crustal carbonate interaction at volcanic arcs^55^. Some periods of global warming in the Earth’s past, notably the PETM, are accompanied by negative δ^13^C excursions of several per mil in the rock record, which could be explained by either massive volcanism^54^ and/or organic carbon release (permafrost and/or methane hydrates)^53^. Although our data show highly negative calcite δ^13^C values, and therefore imply the release of CO_2_ with commensurate highly negative δ^13^C, the bulk gas released from carbonate interaction will always range between the initial carbonate value and the initial carbonate + the CO_2_-calcite fractionation factor (e.g. Δ^13^C_CO2-calcite_ = 3.7 to 2.7‰ at 500–1000 °C^19^), thus driving the δ^13^C of released CO_2_ at volcanoes to relatively high values. A suggested increased volume of crustal carbonate CO_2_ release during PETM^55^ would therefore increase the δ^13^C composition of the global volcanic CO_2_ output above typical mantle values (c.f.^2,56^). Accepting carbonate assimilation in arcs as a contributing factor during past climate perturbations, carbon cycling models would require a much higher input of light carbon than previously thought to balance the isotopically heavier limestone-derived volcanic volatiles to still explain past negative carbon isotope excursions. Although further discussion of carbon cycling modelling is beyond the scope of this paper, we note that the increasingly recognised contribution of limestone-derived carbon to volcanic carbon budgets warrants consideration in carbon cycling models throughout Earth history.

## Methods

### Electron microprobe analysis

Major element composition of minerals, as well as major element, chlorine and sulphur concentrations in groundmass and interstitial glasses and melt inclusions were determined with a JXA 8900 R Electron Probe Microanalyser at the University of Kiel, Germany. Silicate and oxide minerals were analysed with a 15 kV accelerating voltage and 15 nA beam current. Calcite was measured with a 7 µm beam diameter at 15 kV accelerating voltage and a 10 nA beam current to minimise beam damage. Glasses were measured with a 5 µm beam at 15 kV accelerating voltage and a 12 nA beam current. Measurement times were 15 s peak and 7 s background, excluding S, Cl, P, Ba and Sr, which were measured for 60 s and 30 s respectively. Extended counting times of 30 s and 10 s respectively for Fe, Mg and Mn were applied during calcite determination. Na was measured first to minimise alkali migration. Mineral standards were used for calibration and Smithsonian basaltic glass A-99, forsterite 83 USNM2566, plagioclase USNM115900, garnet RV2 USNM 87375, and obsidian ASTIMEX Block SPGLASS7 were used as secondary within run standards to assess accuracy and precision. Errors are less than 3% for major elements and <10% for minor elements.

### Secondary ion mass spectrometry (SIMS) analysis

Sample preparation and *in-situ* isotopic data were acquired at the Edinburgh Ion Microprobe Facility at the University of Edinburgh. The samples were prepared as 3 mm micro-drilled thin section cores pressed into indium, preserving their original textural configuration. A calcite standard (UWC-1) was mounted in epoxy and pressed into the centre of each mount. Thin section MX1 was cut into a 1 inch diameter section with the calcite standard mounted into a hole drilled into the centre of the section. To minimise instrumental bias relative to sample position, each core was mounted to ensure all analyses were within 5 mm of the centre of the mount. All samples were polished after pressing and gold coated.

Oxygen isotope data were acquired with a Cameca ims 1270 ion microprobe, using a ~4 nA primary ^133^Cs^+^ beam. Secondary ions were extracted at 10 kV, and ^16^O^−^ (~4.0 × 10^9^ cps) and ^18^O^−^ (~7.0 × 10^6^ cps) were monitored simultaneously on dual Faraday cups (L′2 and H′2). Each analysis involved a pre-sputtering time of 60 seconds, followed by automatic secondary beam and entrance slit centering and finally data collection in 10 cycles, amounting to a total count time of 40 seconds.

Carbon isotope data were acquired using a ~4 nA beam. Secondary ions were extracted at 10 kV, and ^12^C^−^ (~1.0 × 10^7^ cps) and ^13^C^−^ (~1.0 × 10^5^ cps) were monitored on Faraday cup (L′1) and electron multiplier (EMO). Each analysis involved a pre-sputtering time of 60 seconds, followed by automatic secondary beam and entrance slit centering and finally data collection in 40 cycles, amounting to a total count time of 160 seconds.

To correct for instrumental mass fractionation (IMF), all data were normalised to an internal standard (UWC-1 δ^18^O_SMOW_ 23.3‰, δ^13^C_PDB_ −2.14‰), which was repeatedly measured throughout the analytical sessions. Analyses were made in blocks of 10 followed by 3 to 5 analyses of the standard. The internal precision of each analysis is +/− 0.02‰. The average precision for a typical standard bracket is 0.26‰ for oxygen, and 0.70‰ for carbon (2σ). Each pit was imaged using a scanning electron microscope at Keele University following analysis. Analyses from pits that showed irregularities such as fractures, cavities and mineral overlap were discarded. Instrumental bias due to variations in calcite composition were not considered important as calcite non-CaO concentrations of all samples were <0.30 wt%. All data are reported in standard δ-notation ($\delta =1000(\frac{{R}_{sample}}{{R}_{standard}}-1)\,\textperthousand$ where for example $R=\frac{{\delta }^{18}O}{{\delta }^{16}O}$) relative to SMOW for oxygen and PDB for carbon. 69 paired carbon and oxygen isotope analyses were made, and 8 carbon or oxygen isotope analyses where the calcite was too small for two spots. 75 oxygen and 56 carbon isotope standard analyses were made.

### Modelling

#### Decarbonation

Decarbonation of carbonates produces a decrease in δ^18^O and δ^13^C in the restitic carbonate as heavier isotopes are preferentially removed in the released CO_2_, following the Rayleigh fractionation law^15^:

$${\delta }_{f}-{\delta }_{i}=1000({F}^{(\alpha -1)}-1)$$

where δ_f_ and δ_i_ are final and initial isotopic values, F is the fraction of calcite remaining, and α is the calcite-CO_2_ fractionation factor.

The δ^13^C decrease can be significant, covering more than 10 δ units, but the effect on δ^18^O is less pronounced due to the ‘calc-silicate limit’^15^, a result of newly created silicate minerals representing the major oxygen reservoir. All carbon is released as CO_2_ whilst only ~40% of the oxygen is removed (therefore ${F}_{oxygen}\approx 0.4{F}_{carbon}+0.6$). A typical reaction that exemplifies this is $\mathop{CaC{O}_{3}}\limits^{calcite}+\mathop{Si{O}_{2}}\limits^{silica}=\mathop{CaSi{O}_{3}}\limits^{wollastonite}+C{O}_{2}$. A Rayleigh decarbonation curve following calc-silicate decarbonation at 850 °C is shown in Fig. 3A. This temperature is consistent with the clinopyroxene saturation temperatures calculated from the magmatic skarn xenolith glasses (755–917 °C, Equation 34^57^). This thermometer reproduces experimental low temperature 900 °C carbonate assimilation data^48^ within the published error of 45 °C. Varying temperatures (e.g. ± 200 °C) produces a negligible 1‰ variation on the calculated δ^18^O fractionation curves, and a 3‰ difference δ^13^C at 99% decarbonation (F = 0.01). At 850 °C, the carbon and oxygen isotopic compositions of the released CO_2_ are ~3 and 3.5‰ higher than the calcite^19^, resulting in lowering of calcite isotopic values with increasing decarbonation. The initial calcite C-O isotopic composition is chosen as the highest δ^13^C and δ^18^O values for local limestone^12,25,26^ and additionally an average of the type A calcite compositions, which likely represent unmodified calcite. Decarbonation can alternatively occur via the silicate-absent reaction $\mathop{CaC{O}_{3}}\limits^{calcite}=\mathop{CaO}\limits^{lime}+C{O}_{2}$ (Fig. 3A) which we have additionally modelled (where ${F}_{oxygen}\approx 0.67{F}_{carbon}+0.33$).

#### Calcite-magmatic fluid interaction

This trend is modelled in Fig. 3B, using the mass balance equation of^20^:

$${X}_{C{O}_{2}}\cdot w/r=ln(\frac{{\delta }^{13}{C}_{C{O}_{2}}^{i}+(\Delta -{\delta }^{13}{C}_{cc}^{i})}{{\delta }^{13}{C}_{C{O}_{2}}^{i}-({\delta }^{13}{C}_{cc}^{f}-\Delta )})$$

where ${X}_{C{O}_{2}}$ is the mole fraction of CO_2_ and H_2_O in the fluid phase, *w*/*r* is the fluid/rock ratio, ${\delta }^{13}{C}_{C{O}_{2}}^{i}$ is the initial fluid composition, ${\delta }^{13}{C}_{cc}^{i}$ is the initial calcite composition, ${\delta }^{13}{C}_{cc}^{f}$ is the final calcite composition, and *Δ* is the equilibrium isotope fraction between calcite and CO_2_. Oxygen is modelled using the equivalent formula that lacks the ${X}_{C{O}_{2}}$ term.

The assumed fluid composition is based on the carbon isotope composition of the baseline Merapi fumarole gases (−4.1‰^12^), and oxygen isotopic composition from the estimated primary Merapi magma composition (+6.1‰^58^), coupled with the basalt rock-water fractionation factor^59^ of α = 0.9988114. Although this baseline value may reflect ongoing carbonate interaction at Merapi, using estimated carbon isotope Indonesian mantle values from Krakatau of −6.72 and −6.4‰^60^, or elevated values during eruptive periods of −2.2 to −2.6‰, does not change the overall interpretation of a magmatic fluid presence. We used a XCO_2_ of 0.4, which affects the curvature of the models (concave to straight at unity). We chose this value because of the presence of CO_2_ fluid inclusions and the highly vesicular nature of the xenoliths which is evidence of a strong presence of CO_2_. Interstitial xenolith glasses are Ca-contaminated magmatic glasses, representing a melt that originally contained up to 6 wt % H_2_O^33,61^, allowing for a reduction in XCO_2_. Additionally, the mineral assemblage wollastonite + garnet + anorthite, found in both magmatic skarn sample MX1 and exoskarn sample CS11, require an XCO_2_ < ~0.6 at 100 MPa^37^. This pressure is consistent with the pressure estimated from microthermometry of wollastonite-hosted fluid inclusions (34–94 MPa). Regardless of the exact fluid mole fraction, a magma-carbonate mixing curve cannot reproduce the bulk of the data, in contrast to many worldwide skarns^15,22^.

#### Merapi crustal CO_2_ release

A mass balance model^49^ is utilised to estimate the percentage of the CO_2_ output at Merapi that is crustal derived.

$$ \% carbonate=100\frac{{\delta }^{13}{C}_{fumarole}-{\delta }^{13}{C}_{mantle}}{{\delta }^{13}{C}_{carbonate}-{\delta }^{13}{C}_{mantle}}$$

A mantle carbon isotope value of δ^13^C = −6.5‰ is chosen, based on measurements from nearby Krakatau (−6.7 and −6.4‰), which likely represents a primary Indonesian mantle value^60^. To constrain the crustal carbon output at Merapi, both the averaged Merapi baseline and the 2006 syn-eruptive fumarole δ^13^C values (−4.1‰ and −2.4‰ respectively^12^) are employed in our modelling. Although a δ^13^C value of −4.1 is within some commonly used uncontaminated MORB mantle ranges (e.g. −6 ± 2 ^2^), gas He isotopes indicate that the Merapi gas baseline during quiescence has a crustal carbonate overprint^26^. For this reason, we use −6.5‰ as the primary mantle δ^13^C value. To approximate the δ^13^C values of the carbonate crust underlying Merapi, a range of values are utilised, from −2.2‰^12^ to +3.5‰ (this study). The source of the CO_2_ is assumed to be dominantly crustal and not from subducted sediment on the basis of CO_2_/S_T_ measurements^1^.

To calculate the mass of CO_2_ released by a magma reservoir below Merapi, we followed the calculations of reference^22^,which estimate the amount of CO_2_ released from the aureole around a magma reservoir for a specified reservoir volume, aureole thickness, and decarbonation efficiency. The size of the pre-eruptive magma reservoir below Merapi is poorly constrained, but is known to reside within the carbonate substrata^33,44^. Using the erupted volume from the 2010 paroxysmal eruption of 0.02 to 0.05 km^3^ ^50^ and an average intrusive:extrusive ratio of 5:1^55,62^, we assume an estimated reservoir size of 0.1 to 0.25 km^3^. Thermal and metasomatic aureoles can be extensive in size^63^, so we calculate a range of aureole thicknesses from 5 to 30% of the reservoir width. Aureoles are thermally and chemically gradational, with proximal thermally-affected fully decarbonated and/or assimilated rocks, through metasomatic rocks, to distal marbles, therefore we use a conservative 50% decarbonation efficiency as an average for our aureole calculations. This is in line with a lack of olivine, periclase and other magnesian phases in the xenoliths, which implies the carbonate protolith at Merapi is highly likely to be limestone, which has a lower decarbonation efficiency than dolomite^55^.

## Supplementary information

Supplementary Discussion, Dataset and Figure

## Data Availability

The authors declare that all relevant data are available within the article and its supplementary information files.
