# Supplementary material for: Crustal CO2 contribution to subduction zone degassing recorded through calc-silicate xenoliths in arc lavas
Source: Sci Rep. 2019 Jun 19;9:8803. doi: 10.1038/s41598-019-44929-2 (PMC6584678; doi:10.1038/s41598-019-44929-2)
Supplement: Supplementary file 1 — Supplementary Discussion, Dataset and Figure [file 41598_2019_44929_MOESM1_ESM.pdf]

Crustal CO_2 contribution to subduction zone degassing recorded through calc-silicate xenoliths in arc lavas

Supplementary Material

Sean Whitley* [1]

Ralf Gertisser [1]

Ralf Halama [1]

Katie Preece [2]

Valentin Troll [3,4]

Frances Deegan [3]

[1] School of Geography, Geology and the Environment, Keele University, Keele, ST5 5BG, UK

[2] Department of Geography, College of Science, Swansea University, SA2 8PP, UK

[3] Section for Mineralogy, Petrology and Tectonics (MPT), Department of Earth Sciences, Uppsala University, 752 36 Uppsala, Sweden

[4] Faculty of Geological Engineering, Universitas Padjajaran (UNPAD), Bandung, Indonesia

Corresponding authors: Sean Whitley; s.o.whitley@keele.ac.uk

Ralf Gertisser; r.gertisser@keele.ac.uk

Primary Calcite Justification

Calcite is a common secondary alteration product in a range of rock types, therefore the origin of the calcite must be considered before interpreting the data. We consider the xenolith calcite to be primary relics of calcite, as opposed to secondary hydrothermal calcites, for the following reasons:

Firstly, the analysed xenoliths are exceptionally fresh, with minor red oxidation of Fe-Ti oxides observed in sample CS11 only. Hydrous phases, either primary or as secondary replacement, are not observed in any of the samples studied, implying that fluid overprint was not a factor. Glass within the xenoliths is optically clear, lacking any devitrification or alteration. Typically void-filling, low-temperature secondary phases such as zeolites are not observed. Calcite is, in turn, only found within the xenolith cores, except for type A calcites in sample CS17 where calcite can be present in glass-hosted vesicles surrounding the wollastonite dominated core zone. These latter calcite crystals do not demonstrate a geode-like internally zoned texture that would suggest secondary infill of vesicles[1], but instead have a globular habit, more indicative of a quenched previously molten calcite[2,3]. In addition, secondary calcite precipitation would be expected to distribute calcite throughout the sample, wherever there may be vesicles or fractures. Type C-melt like calcite does not follow fractures or cleavage, and does not crosscut minerals, but follows the crystal boundaries.

The mineral assemblages in the xenoliths exclusively indicate high magmatic or metamorphic temperatures (>400°C) and are inconsistent with a low-temperature or hydrothermal overprint. Wollastonite is unstable below ~400°C[4], therefore interaction

with meteoric water or hydrothermal fluids would be expected to cause alteration, which is not observed. Melt-like calcite (Type C) is consistent with a high temperature origin due to the equilibrium assemblage containing fluorite and cuspidine ($Ca_4Si_2O_7F_2$). Fluorine and fluorite can increase the stability of molten calcite down to 880°C[5], similar to temperatures estimated from the interstitial xenolith glass (755-917°C) using the glass thermometer equation 34[6]. Type E calcites are surrounded by a prominent reaction rim containing high temperature mineral assemblages; Spurrite ($Ca_5(SiO_4)_2CO_3$) is stable above 430°C at low CO_2 partial pressures[7], and 700-1000°C at higher XCO_2[8]. Wollastonite, grossular and plagioclase found on the outer edge of the reaction rim are stable between ~510-890°C[9].

Chemically, significant $\delta^{13}C$ depletions can occur from contamination by organic material, as has likely occurred on the surface of some Merapi basaltic andesite whole-rock samples that interacted with burning surface vegetation[10,11]. Measuring these fresh calcites *in-situ* avoids possible surface contamination.

Finally, the remote possibility that the investigated calcites represent a carbonatite magma (i.e. mantle derived carbonate melt) is also unlikely due to the high $\delta^{18}O$ values greatly exceeding mantle values, instead overlapping marine carbonate values. They additionally lack the high SrO interpreted to represent primary carbonatite calcite[12], having SrO concentrations (≤0.08 wt%) closer to local limestone near to Merapi (0.01 wt%;[13]). We therefore conclude the calcites to be derived from sedimentary carbonate in the direct basement to Merapi volcano.

References

1. Lavoie, D. Carbonate botryoids in Lower Devonian amygdaloidal basalts; evidence for precipitation of high-magnesium calcite from heated and volcanic CO_2-buffered marine waters. *J. Sediment. Res.* **65**, 541–546 (1995).

2. Lucido, G., Nuccio, P. M., Leone, G. & Longinelli, A. Amygdaloidal basalts: Isotopic and petrographic evidence for non-diagenetic crustal source of carbonate inclusions. *Tschermaks Min. Pete Mitt.* **27**, 113–128 (1980).

3. Bailey, D. K. & Kearns, S. New forms of abundant carbonatite–silicate volcanism: recognition criteria and further target locations. *Mineral. Mag.* **76**, 271–284 (2012).

4. Greenwood, H. Wollastonite: stability in H_2O-CO_2 mixtures and occurrence in a contact-metamorphic aureole near Salmo. *British Columbia, Canada.American Mineralogist* **52**, 1669–1680 (1967).

5. Jago, B. C. & Gittins, J. The role of fluorine in carbonatite magma evolution. *Nature* **349**, 56 (1991).

6. Putirka, K. D. Thermometers and barometers for volcanic systems. *Rev. Mineral. Geochem.* **69**, 61–120 (2008).

7. Henmi, &. H., C. Synthesis of spurrite and tilleyite at low CO_2 partial pressure. *Mineralogical Journal* **9**, 106–110 (1978).

8. Tuttle, O. F. & Harker, R. I. Synthesis of spurrite and the reaction wollastonite+calcite ⇌ spurrite+carbon dioxide. *Am. J. Sci.* **255**, 226–234 (1957).

9. Tracy, R. J. & Frost, B. R. Phase equilibria and thermobarometry of calcareous, ultramafic and mafic rocks, and iron formations. in *Rev. Mineral. Geochem.* (ed. Kerrick, D. M.) **26**, 207–289 (Mineralogical Society of America, 1991).

10. Donoghue, E., Troll, V. R., Schwarzkopf, L. M., Clayton, G. & Goodhue, R. Organic block coatings in block-and-ash flow deposits at Merapi Volcano, central Java. *Geol. Mag.* **146**, 113–120 (2009).

11. Troll, V. R. *et al.* Crustal CO_2 liberation during the 2006 eruption and earthquake events at Merapi volcano, Indonesia. *Geophys. Res. Lett.* **39**, (2012).

12. Barker, D. Origin of cementing calcite in 'carbonatite' tuffs. *Geology* **35**, 371–374 (2007).

13. Chadwick, J. Crustal processes in volcanic systems: case studies from Northern Ireland, New Zealand, and Indonesia. (Trinity College Dublin, 2008).

Supplementary Tables

Supplementary Table 1 Phases identified within the magmatic and exoskarn xenoliths at Merapi.

Magmatic Skarns	Exoskarns
Rock-Forming Phases	
Wollastonite	Wollastonite
Clinopyroxene (diopside-hedenbergite)	Clinopyroxene (Ca-Tschermaks-rich)
Anorthite	Anorthite
Glass	Grossular-Andradite Garnet
	± Gehlenite
	± Spinel
	± Quartz
	± Calcite
Accessory Phases	
Calcite	Anhydrite
Clinopyroxene (Ca-Tschermaks-rich)	Pyrrhotite
Grossular-andradite-schorlomite garnet	Ellestadite
Wadalite-like phase	Cuspidine
Cuspidine	Spurrite
Gehlenite	Larnite
Pyrrhotite	Xenotime
Cubanite	Hematite
Baryte	Monazite
Anhydrite	
Apatite	
Ellestadite	
Ferro-bustamite	
Titanite	
Perovskite	
Magnetite	

Supplementary Table 2 Electron microprobe major element analyses of texturally distinct calcite types within the calc-silicate xenoliths. Type A - glass-hosted calcites were not analysed by microprobe, but show negligible concentrations of major elements excluding CaO in SEM-EDS analysis. Results are reported as averages with standard deviation in parentheses.

	Type B-Interstitial	Type C-Melt-like	Type D-Inclusions	Type E-Residual
wt%	(n=1)	(n=5)	(n=2)	(n=10)
FeO	0.02	0.06 (0.04)	0.17 (0.08)	0.07 (0.06)
MnO	0	0.12 (0.08)	0.08 (0.18)	0.12 (0.09)
MgO	0.05	0.06 (0.03)	0.05 (0.01)	0.05 (0.02)
CaO	58.68	55.1 (0.59)	54.54 (0.17)	55.48 (0.64)
SrO	n.d	0.02 (0.01)	0 (0)	0.06 (0.01)
BaO	n.d	0.01 (0.02)	0.02 (0.03)	0.01 (0.01)
Total	58.75	55.37 (0.52)	54.76 (0.25)	55.79 (0.53)

Supplementary Table 3 C and O isotopic compositions of calcite in calc-silicate xenoliths of Merapi volcano. 2σ errors are typically 0.4 ‰ for oxygen and 0.8 ‰ for carbon.

Sample	Calcite Type*	Rock Type	$\delta^{18}O$ (‰)	$\delta^{13}C$ (‰)
CS17	A	Magmatic	+23.8	-3.7
CS17	A	Magmatic	+22.7	-4.2
CS17	A	Magmatic	+24.0	+1.8
CS17	A	Magmatic	+21.4	+1.8
CS17	A	Magmatic	+22.3	+0.4
CS17	B	Magmatic	+21.6	-6.8
CS17	B	Magmatic	+21.3	-0.6
CS17	B	Magmatic	+20.5	-6.4
CS17	B	Magmatic	+20.5	-6.5
CS17	B	Magmatic	+24.3	-13.5
MX3	B	Magmatic	+24.2	-29.3
MX3	B	Magmatic	+23.5	-29.1
MX3	B	Magmatic	+23.6	-28.3
MX3	B	Magmatic	+24.1	-28.3
MX3	B	Magmatic	+25.6	-22.6
MX5	B	Magmatic	+23.8	-22.5
MX5	B	Magmatic	+20.5	-14.9
MX5	B	Magmatic	+24.9	-19.3
MX5	B	Magmatic	+24.0	-18.8
MX5	B	Magmatic	+24.0	-19.7
MX5	B	Magmatic	+23.5	-21.5
MX5	B	Magmatic	+23.1	-25.1
MX5	B	Magmatic	+22.9	-22.5
MX5	B	Magmatic	+23.8	-22.7

Supplementary Table 3 continued

MX1	C	Magmatic	+20.1	-13.6
MX1	C	Magmatic	+14.0	-12.4
MX1	C	Magmatic	+21.5	-8.1
MX1	C	Magmatic	+22.0	-6.3
MX1	C	Magmatic	+23.0	-0.1
MX1	C	Magmatic	+23.1	-2.7
MX1	C	Magmatic	+19.3	-3.5
MX1	C	Magmatic	+23.0	-0.8
MX1	C	Magmatic	+19.9	-8.7
MX1	C	Magmatic	+17.9	-18.5
MX1	C	Magmatic	+19.6	-0.1
MX1	C	Magmatic	+17.8	+3.3
MX1	C	Magmatic	+18.9	+3.5
MX1	C	Magmatic	+9.9	-15.9
MX1	D	Magmatic	+17.9	-4.4
MX1	D	Magmatic	+14.6	-6.4
MX1	D	Magmatic	n.a.	-9.6
MX5	D	Magmatic	+17.6	-13.8
MX5	D	Magmatic	+17.0	-14.9
CS11	E	Exoskarn	+15.7	-12.9
CS11	E	Exoskarn	+15.5	-14
CS11	E	Exoskarn	+19.0	-8.3
CS11	E	Exoskarn	+18.6	-8.4
CS11	E	Exoskarn	+18.6	-7.8
CS11	E	Exoskarn	+18.6	-7.8
CS11	E	Exoskarn	+18.7	-7.4
CS11	E	Exoskarn	+15.0	-9.0
CS11	E	Exoskarn	+15.0	-11.0
CS11	E	Exoskarn	+14.9	-8.5
CS11	E	Exoskarn	+15.2	-9.8
CS11	E	Exoskarn	+15.1	-9.1
M13-02	E	Exoskarn	+14.0	-12.1
M13-02	E	Exoskarn	+13.9	-11.7
M13-02	E	Exoskarn	+14.0	-12.1
M13-02	E	Exoskarn	+14.3	-11.8

Supplementary Table 3 continued

M13-02	E	Exoskarn	+14.3	-10.5
M13-02	E	Exoskarn	+16.5	-4.9
M13-02	E	Exoskarn	+16.7	-4.9
M13-02	E	Exoskarn	+16.5	-4.6
M13-02	E	Exoskarn	+16.6	-5.0
M13-02	E	Exoskarn	+16.8	-4.7
M13-02	E	Exoskarn	+17.0	-5.5
M13-02	E	Exoskarn	+17.2	-5.1
M13-02	E	Exoskarn	+17.0	-5.9
M13-02	E	Exoskarn	+18.4	-5.6
M13-02	E	Exoskarn	+15.8	-5.7

* Calcite type: A = Glass hosted; B = Interstitial; C = Melt-like; D = Inclusion; E = Residual

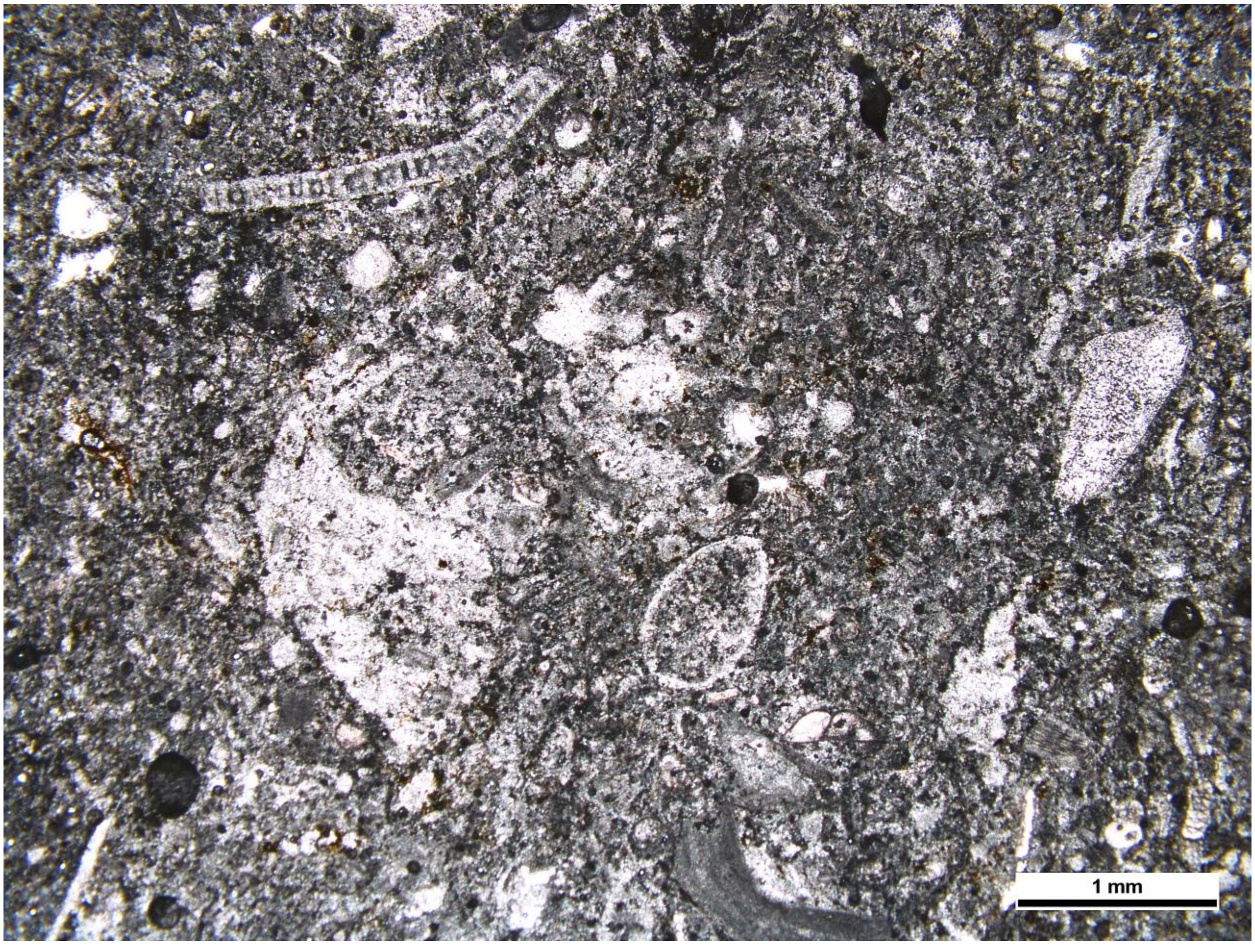

Supplementary Fig. 1 Local limestone sampled from Parangtritis. Thin section imaged in transmitted light.
